# On opportunities and challenges of large multimodal foundation models in education

**DOI:** 10.1038/s41539-025-00301-w

**Published:** 2025-02-26

**Authors:** Stefan Küchemann, Karina E. Avila, Yavuz Dinc, Chiara Hortmann, Natalia Revenga, Verena Ruf, Niklas Stausberg, Steffen Steinert, Frank Fischer, Martin Fischer, Enkelejda Kasneci, Gjergji Kasneci, Thomas Kuhr, Gitta Kutyniok, Sarah Malone, Michael Sailer, Albrecht Schmidt, Matthias Stadler, Jochen Weller, Jochen Kuhn

**Affiliations:** 1 Chair of Physics Education, Faculty of Physics, Ludwig-Maximilians-Universität München, Munich, Germany; 2 Chair of Education and Educational Psychology, Ludwig-Maximilians-Universität München, Munich, Germany; 3 Institute of Medical Education, University Hospital, Ludwig-Maximilians-Universität München, Munich, Germany; 4 Chair for Human-Centered Technologies for Learning, Technical University of Munich (TUM), Munich, Germany; 5 Chair for Responsible Data Science, Technical University of Munich (TUM), Munich, Germany; 6 Faculty of Physics, Ludwig-Maximilians-Universität München, Munich, Germany; 7 Chair of Mathematical Foundations of Artificial Intelligence, Ludwig-Maximilians-Universität München, Munich, Germany; 8 Department of Physics and Technology, The Arctic University of Norway, Tromsø, Norway; 9 DLR - German Aerospace Center, Oberpfaffenhofen, Germany; 10 Department of Education, Campus A 4.2., Saarland University, Saarbrücken, Germany; 11 Chair of Learning Analytics and Educational Data Mining, University of Augsburg, Augsburg, Germany; 12 Chair of Human-Centered Ubiquitous Media, Ludwig-Maximilians-Universität München, Munich, Germany

**Keywords:** Education, Human behaviour, Society, Sociology, Interdisciplinary studies

## Abstract

Recently, the option to use large language models as a middleware connecting various AI tools and other large language models led to the development of so-called large multimodal foundation models, which have the power to process spoken text, music, images and videos. In this overview, we explain a new set of opportunities and challenges that arise from the integration of large multimodal foundation models in education.

## Introduction

The advent of large language models (LLMs) like ChatGPT-3.5, developed by OpenAI, marked a pivotal moment in the potential of artificial intelligence (AI) in education. This manuscript builds upon prior overviews of LLMs in education, which outlined the transformative potential and inherent challenges of integrating LLMs into educational environments^1–4^. With the introduction of ChatGPT-3.5, it appeared as though we had reached the pinnacle of LLMs. This was a momentous advancement in the application of AI. However, the landscape of AI has proven to be ever-evolving with increasingly short innovation cycles. The recent emergence of large multimodal foundation models (LMFMs), such as ChatGPT-4-Turbo by OpenAI or Gemini by Google, has reshaped our previous perceptions, bringing to the table a fresh set of opportunities and challenges for education. This overview aims to delve deeper into the opportunities and challenges presented by these advanced LMFMs, such as ChatGPT-4-Turbo and Gemini, shedding light on the future of AI in education.

Historically, the integration of AI in education has been a gradual but impactful process. From the early adoption of systems that adapt the sequence of tasks and learning content to learners’ understanding with early intelligent tutoring systems^5^ to more learner-centered systems that include natural language processing^6^, the educational landscape has been reshaped^7^. The development of the Generative Pretrained Transformer (GPT) and Bidirectional Encoder Representations from Transformers (BERT) marked a significant leap forward in the processing of natural language texts and – by implication – the processing of learners’ and teachers’ inputs and consequent responses. These models, trained on extensive text data, showcased an unprecedented ability to generate human-like text, answer complex questions, classify teachers’ written reflections and facilitate very high learner-centered interactive learning experiences^8,9^. The release of ChatGPT-3.5 especially sparked numerous empirical studies demonstrating the wide variety of opportunities for education^3^. For instance, it can be used to support teachers in generating course material^10,11^, to detect students’ errors during experimentation^12^, to enhance peer feedback processes^13^, to augment empirical data in education^14^ and to provide various types of formative feedback to enhance self-regulated learning^15^. At the same time, there are also numerous challenges and concerns, such as the risk of overreliance by students on the output of an LLM without critically reflecting on it, the difficulty for teachers to identify texts that were created by an LLM rather than by students. There are concerns about the reliability and accuracy of the output of the LLM, potential inherent biases, and the protection of students’ and teachers’ personal data^1^.

A few months after the release of ChatGPT, it became available via an application programming interface (API). This allowed for its integration into custom-built applications with specific preprompting that allow the assignment of tasks and roles to the LLM^15^. As an important step, Shen and colleagues demonstrated that large language models can be effectively used as a middleware to communicate between different AI models by using language as the universal interface of every AI model^16^. In this way, the vast amount of developed AI tools can be effectively integrated into advanced LLMs, provided the LLM is fine-tuned to the specifications of the AI tools to generate correct inputs. This effective communication between a LLM and different AI tools led to the development of LMFMs, which not only allow an input and output via written text, but also via spoken text, images or videos^17^. Among these advanced LMFMs are ChatGPT-4-Turbo and Gemini. In comparison to the first version of ChatGPT-3.5, these models represent a significant advancement in this field due to its multimodality, boosted accuracy, faster response times, and more nuanced understanding of context and subtleties in language.

The potential opportunities of LMFMs in education are manifold. Their advanced capabilities can revolutionize personalized learning, enabling tailor-made educational experiences that adapt to individual student needs and learning preferences. The enhanced understanding and response mechanisms can aid in creating more engaging and interactive multimodal content, thus potentially increasing student motivation and participation in learning activities. Moreover, their ability to process and generate vast amounts of information in real time can serve as a powerful tool for research on learning and teaching processes.

However, with these opportunities come additional significant challenges. The reliance on AI-supported education raises questions about the accuracy and reliability of the information provided by these systems. The potential for inherent biases in the AI algorithms also remains a critical issue with LMFMs, and this issue may be intensified due to the complexities arising from processing multiple input modalities. Furthermore, the integration of such advanced technologies in educational settings necessitates a reevaluation of teaching methods and curricula. It also requires the development of new competencies among educators and students alike because these tools now allow for much more interactive learning and formative feedback, thus allowing a shift from a focus on declarative knowledge to facilitating complex skills. This is a crucial step towards the democratization of education, ensuring that learning opportunities are accessible and equitable, making it more likely now for illiterate people to use AI without even being able to read and write. Yet, the issue of the digital divide and accessibility remains a concern, as not all educational institutions may have the resources to implement and maintain such advanced technology effectively^1^.

The following sections of this manuscript will explore how LMFMs can enhance various aspects of education, such as personalized learning, student engagement, and educational content and application creation, and they will critically examine the potential drawbacks and limitations of LMFMs in education, including issues related to accuracy, bias, and the need for human oversight. Finally, the ‘Mitigation Strategies’ section will propose strategies to overcome these challenges, ensuring that the integration of LMFMs in educational settings is done responsibly, ethically, and effectively. This work aims to provide a comprehensive overview of the landscape of LMFMs in education, with a specific focus on the latest advancements represented by LMFMs, thereby contributing to the ongoing discussion about the role of AI in shaping the future of education.

## Opportunities

### Opportunities for learners

Personalized learning assistants can have multiple benefits for learners. They have the potential to enhance students’ productivity and foster motivation^18^. ChatGPT, for instance, has been favorably received by students^19,20^. Furthermore, the advanced context understanding of LMFMs could potentially alleviate concerns regarding the accuracy of LLM outputs, due to their enhanced precision. Additionally, the integration of LLMs in an API allows efficient preprompting including the provision of an example solution. In this way, LLMs are aware of the correct solution and can guide the learner towards it without generating hallucinations^15^. In the context of LLMs, “hallucination" refers to the generation of information that appears plausible but is factually incorrect. LMFMs offer a range of options for learners, as recent research shows. Fauzi and colleagues discuss the role of GPT in stimulating contextual learning strategies, thereby enhancing metacognitive abilities^18^. This aspect is important for the development of self-regulated learning skills. In addition, the opportunity to build personal learning assistants using LMFMs promotes generative learning^21^, where learners actively participate in the design of their learning tools, which may lead to higher engagement, better understanding, and retention. Furthermore, educators can now create customized LMFM applications with pre-defined prompts, design content based on their needs, the subject and teaching style by providing relevant domain-specific content to the fine-tuned LMFMs which can expedite learning through such personalized learning assistants. In this way, the specially created multimodal learning assistants can be individualized and offered on a large scale in the classroom or school setting. Learners can therefore benefit from using teacher-recommended Chatbots to boost their learning process.

Particularly, LMFMs have the capacity to facilitate a range of learning mechanisms and provide support to students across a diverse array of activities, as outlined in the following:

- **Multimedia-effect**: The capabilities of LMFMs, especially their abilities to process and generate images suggest a more comprehensive learning experience. It is already known from multimedia research that text in combination with images leads to better understanding than text alone^22^. This is particularly beneficial for complex or abstract concepts, where visual representations can provide additional clarity. In this line, LMFMs may enhance formative feedback by providing visual representation along with written feedback to better illustrate learners’ difficulties^23^.
- **Barrier-free interaction**: Furthermore, LMFMs can play a crucial role in supporting students with disabilities by allowing a barrier-free interaction via speech-to-text, image-to-text, text-to-image, and text-to-speech functionalities. These features make educational content more accessible, especially for students with visual or auditory impairments or with difficulties in writing or reading. Similarly, students can also write or speak in their mother tongue if they are unfamiliar with the local language, and the text is translated into the local language. These features can also be beneficial in language learning, where the model can act as a conversational partner, offering real-time interaction and feedback^24^. It could help learners develop their language skills in a more natural and immersive environment, simulating real-life conversations. Similarly, LMFMs may enrich history lessons by enabling role-playing^25^ and acting as conversational partners such as historically important characters like Napoleon, Abraham Lincoln, or others. These features enable a more inclusive learning environment where all students have equal opportunities to engage with and benefit from the educational materials. Additionally, the adaptability of GPTs in customizing learning experiences can help address individual learning needs in a diverse student population with varying abilities and learning preferences^26^.
- **Learning support during the generation of images**: Another significant aspect is the integration of visual transformer models such as DALL-E^27^, which enables the creation of visual outputs based on text inputs. LMFMs have also demonstrated the ability to understand the content of images, thereby offering the opportunity for students to receive learning support during generation through real-time scaffolding and feedback on their drawings. This may prove useful in providing multi-faceted feedback and in subjects where visual representation is key. For instance, in arts and creative subjects, it can be used to teach design and reflection, allowing students to explore different visual representations and interpretations of their ideas^28^. In several other fields of education, the generation of visual representations, such as graphs or schemas, is a common exercise^29^. Here, LMFMs may provide real-time feedback to students while generating graphs.
- **Learning support during the generation of handwritten text, mathematical solutions, and physics equations**: LMFMs have demonstrated the ability to even process images or plots that contain handwritten text, experimental results, function graphs or mathematical equations to provide feedback to students in real-time^30^. In this way, they may not only support students in solving maths problems but also provide feedback during the solution of physics problems^31^.
- **Representational competence training**: Several authors point out that the integration of visual representations in the learning material can enhance learning^32,33^. To effectively learn from visual representations, Edelsbrunner and colleagues could show that students need to possess representational competence that allows them to extract information from visual representations and interpret them^34^. However, Rau points out that students often face a representational dilemma, which means that they do not possess the necessary representational competence during learning, so they need to acquire it during the learning of domain-specific content^33^. State-of-the-art LMFMs allow the explanation of images, translation of data to other representations and can also explain how to extract information from representations to students. In this way, they may enable an effective representational competence training. In this regard, Polverini and Gregorcic showed that ChatGPT-4-Turbo is able to solve tasks with line graphs in physics with a similar overall score as high school students^35^.
- **Animation of phenomena**: Animations can help learners to understand a number of phenomena if they follow certain principles^36^. With effective preprompting, these animation principles could be integrated into learning environments and provide learners with customized animations for their needs.
- **Targeted support during experimentation**: Performing experiments and collecting experimental data can be tedious for learners, often with limited learning gains^37^. Here, LMFMs perform tasks that are not part of the learning goal, for instance to generate artificial measurement data and visualize it at the same time to offer learners a tangible intuition of factual relationships and to test physical laws. Similar to other digital media, they are able to take over certain learning-relevant cognitive activities during experimentation to effectively reduce extraneous cognitive load and free up cognitive resources to focus on achieving the learning goals^38^. Moreover, LMFMs can analyze images of experimental setups and provide personalized support as students are setting up the experiment.
- **Targeted support during code generation**: The advent of APIs has facilitated the development of tools like CodeHelp, which leverage the power of LLMs as discussed by Liffiton et al.^39^. The capabilities of LMFMs further enhance this process. LMFMs enable the visualization of class and method dependencies or flow diagrams in a program. Hence, LMFMs can help create more accessible and engaging learning environments, especially in complex areas such as programming languages. These functionalities are not only for secondary school students, but the multimodality also allows more advanced students in higher education to access learning content with greater ease due to the multimodal nature of these models.
- **Creation of customized LMFM-based learning tools**: In addition, students are now able to create their own customized learning tools based on LMFMs just by verbally describing the tasks of the tool without the need for programming. Such activities, like creating personal learning assistants using LMFMs, promote generative learning, where learners actively participate in the design of their learning tools and digital learning environments, which may lead to a higher engagement, better understanding, and retention.
- **Interpretation of behavioral and physiological data**: As mentioned above, LMFMs understand a variety of inputs including physiological and behavioral data. Therefore, in comparison to conventional LLMs that can provide scaffolding support and feedback based on students’ inputs, LMFMs are able to analyze, visualize, and interpret students’ behavioral and physiological data and provide actionable feedback and guidance to learners. For instance, they can visualize these data in a dashboard for students to support self-reflection and identify the most efficient approaches for learning.
- **Support during collaborative learning**: With advanced context understanding, LMFMs can facilitate collaborative learning through peer feedback models. Bauer and Greisel et al.^13^ suggested a model which uses natural language processing to support peer feedback in digital learning environments. This model describes learners’ activities and textual products, and introduces a scheme to foster the peer feedback process. LMFMs can thus enhance understanding, promote active engagement, and lead to a more enriching learning experience, enhancing metacognitive abilities and self-regulated learning during collaborative learning.
- **Enhancing explainable user interfaces**: Explainable AI in education is employed to make system decisions understandable and verifiable, highlighting, for instance, the involvement and significance of specific features leading to actionable insights^40^. Explainable User Interfaces (XUIs) aim to bring this level of transparency directly to users, presenting system decisions in a clear and interactive manner to enhance users’ agency and understanding. While XUIs hold substantial promise, particularly for applications like explainable learning analytics, the education domain is yet to fully adopt and optimize these interfaces. LMFMs provide potential for enhancing XUIs to better accommodate a wide spectrum of teachers and learners. Traditionally designed with adult users in mind (e.g.,^41^), XUIs must evolve to meet the diverse cognitive and developmental needs of users. The integration of LMFMs could potentially enable more adaptable and varied forms of information representation. Exploring the synergy between LMFMs and XUIs represent a promising direction for making AI in education more inclusive and effective.
- **Educational support in developing countries**: In areas in the world where expert teachers and high-quality education are scarce, applications based on LMFMs may contribute to closing the educational gap, enhancing the overall educational level and providing a diverse multimodal learning experience.

In sum, *LMFMs could make complex concepts more accessible, adapt to different learning preferences, and improve the approach learners use to tackle a problem or task. Combining personalization, contextual support, creative engagement and multimodal content, this multi-faceted approach illustrates the potential of LMFMs to improve the educational landscape*.

### Opportunities for teachers and educators

The use and integration of LMFMs in the classroom presents various new opportunities for educators. Similar to students, teachers may benefit from the seamless interaction with an LMFM and its user-friendliness, which enhances accessibility and ease of use. Combined with the option to create customized applications based on LMFMs that include multi-modal input and output, educators from diverse fields are now able to tailor and employ generative AI tools according to their specific instructional needs. Consequently, this advancement may offer benefits to teachers inside and outside of the classroom.

#### For lesson planning and classroom activities

LMFMs present multiple opportunities. With the ability to create customized tools based on LMFMs and visualize content, educators could have a tool at their disposal to conceptualize and structure their teaching strategies more effectively. This capacity for lesson planning integrates with learning task design, where up-to-date database and multi-modal task design capabilities offer a nuanced approach to educational content creation. The LMFMs’ up-to-date database and access to the internet are beneficial in subjects like social sciences and politics, providing educators with current and historical context. Simultaneously, the ability to design multimodal tasks complements these contextual advantages. By employing an LMFM’s capability to create learning activities that, for instance, include visual elements or symbolic representations, educators may make educational content more accessible and stimulating for learners^42,43^.

#### Exploration of new teaching methods

In addition, the technology may empower educators who are eager to explore new teaching strategies within traditional classroom settings. The introduction of executable code allows teachers to act as developers, who are likely to create bespoke learning experiences that cater to individual student needs, potentially making education more personalized and effective^44,45^. One example is learning environments, such as flipped classroom models and blended learning scenarios, which more strongly incorporate elements of self-regulated learning compared to traditional classroom settings^46,47^. Here, it is plausible that LMFMs will provide resources and support that complement students’ independent learning phases, subsequently enriching the in-class teaching experience^48^. Such environments may be particularly relevant to the development of competencies for higher-order thinking skills. These often demand contextualized problem sets, open-ended problem formulation and discussion^49^, requiring teachers to use highly differentiated instructions. In this way, the use of LMFMs to design tasks that stimulate critical thinking and provide individualized feedback could be a valuable asset in reducing teacher workload (cf.^50^).

#### For extracurricular work and professional development

LMFMs may serve as a versatile tool for educators. In extracurricular contexts, they may act as personalized assistants, streamlining administrative tasks such as scheduling, email management, and document preparation. By automating these tasks with personalized GPTs, educators may reallocate their time towards more critical aspects of teaching and curriculum development. Furthermore, it is likely that LMFMs aid in curriculum design by suggesting tailored resources and visualizations, activities, and assessment methods, aligned with specific learning objectives and student needs. Moreover, LMFMs may provide feedback on teaching strategies based on current educational trends.

#### Creation of simulations of common classroom practices

Chernikova and colleagues have demonstrated in a meta-analysis that simulations of professional situations, such as interactions in a classroom for teachers, can be a beneficial tool during teacher training as they expose teachers to authentic problems in their fields^51^. Similarly, LLMs have demonstrated that they convincingly act as students with specific difficulties in physics^14^. Therefore, LMFMs have the potential to authentically simulate multimodal interactions with students with specific difficulties. Additionally, they may systematically support the teachers in simulation environments by automatically selecting and modifying representations of practice, called representational scaffolds^52^.

#### Support of multimodal assessment creation and grading

Prior research demonstrated that LLMs can effectively support teachers during the creation of assessment tasks in physics of comparable quality to textbooks^10^ or during the assessment of experimentation errors in chemistry education^12^. By extension, LMFMs may also support teachers in creating multimodal assessment tasks and provide an initial correction of students’ solutions in the form of handwritten text, handwritten mathematical solutions, or students’ drawings.

#### Creation of teacher dashboards

As mentioned above, LMFMs can analyze student performance data and behavioral data of teachers. LMFMs may create data visualizations and create dashboards for teachers to provide an overview of class performance and give effective feedback to teachers^53,54^. Based on this analysis, teachers may adapt their teaching strategy and identify the most efficient approaches used in the classroom.

### Opportunities for (educational) researchers

The transition to LMFMs represents a breakthrough not only for educational research, but also across diverse academic disciplines.

#### Advanced data analysis

LMFMs introduce advanced features for data analysis, such as data cleaning and data processing^55,56^, and corresponding visualizations. These features significantly streamline the data analysis process, allowing for more experiments or studies to be conducted in the same timeframe. Here, the capabilities go beyond basic analysis, handling complex statistical tasks and processing large data sets. Furthermore, the conversational nature of ChatGPT-4 transforms the data analysis tool into more than just a mechanism for cleaning, evaluating, and presenting data. It serves as a guide, engaging in a dialogue with the researcher, elucidating the coding behind each analysis, and rationalizing every decision made. This interaction enables the tool to adapt to the researcher’s criteria and preferences, fostering a collaborative environment. These enhancements pave the way for more nuanced, accurate, and efficient research.

#### Literature reviews

LMFMs often represent a remarkable advance in contextual understanding and language generation. They outperform conventional LLMs, such as ChatGPT-3.5, in terms of accuracy and depth^57^, which allows for more efficient processing of large volumes of textual data. Additionally, the combination of direct access to internet resources and enhanced contextual understanding facilitates comprehensive literature reviews and in-depth thematic analysis with unprecedented efficiency. Moreover, by elevating the accuracy of content generation, advanced LMFMs significantly contribute to the formulation of research texts, aiding in the creation of draft papers and proposals.

#### Advanced graphical data interpretation

As mentioned above, LMFMs exhibit the potential to decipher and interpret the context and components within figures^35,57,58^, assisting researchers in comprehending the underlying narrative and conveying the key message encapsulated in visual representations. Additionally, they have the capacity to provide guidance on optimizing figure design for clarity and comprehension by audiences. This multifaceted assistance signifies a promising trajectory in which AI can augment researchers’ abilities to interpret and communicate complex information across diverse modalities.

#### Creation of multimodal assessment and learning material

LMFMs do not only benefit teachers by aiding in the creation of multimodal assessment and learning materials, but they also may prove useful to researchers. These materials can be utilized in empirical research studies. Additionally, they can assist researchers in categorizing and grading handwritten text or math solutions, thereby facilitating the analysis of studies.

#### Advanced research opportunities

Prior to the release of LMFMs, the development of intelligent tutoring systems was cost- and time-intensive, and required skilled personnel. Therefore, the development of tutoring systems was not common, and research in this area was not widely possible. Now, the opportunity to develop customized LMFM apps (‘GPTs’^59^ in case of ChatGPT-4-Turbo) empowers researchers to create and test customized intelligent tutors, which can also be readily shared with a global audience. This grants teachers and students swift access to state-of-the-art research tools, fostering an environment where cutting-edge advancements seamlessly integrate into educational practices. Additionally, its robust speech-to-text and text-to-speech processing capabilities enable studies where learners can interact with computers in a manner that more closely mirrors the dynamics and interactivity of a traditional classroom setting.

#### Support for diverse, multilingual and interdisciplinary research teams

LMFMs, designed for multilingual tasks, are likely to provide valuable assistance to diverse, interdisciplinary, and multilingual research teams by promoting effective communication within diverse teams. Special terms or methodological approaches in certain disciplines may be rephrased and explained in simple terms to researchers from other disciplines. Similarly, LMFMs can provide a real-time translation from spoken text to another preferred language enabling collaboration between research teams that do not speak a common language. This ability to bridge language and professional barriers may significantly enhance collaboration and productivity in research teams.

Within the realm of educational research specifically, the new possibilities of LMFMs are manifold. Their proficiency in understanding multiple languages and representations offers a promising avenue for bridging the communication gap between researchers and students from different parts of the world.

### Opportunities for developers of educational applications

#### Development of customized intelligent learning applications

Since several years, intelligent tutoring systems (ITS) have been developed and effectively integrated into learning environments to provide enriched personalized learning experiences, for instance, by offering tailored feedback and guidance^60,61^. However, their development and integration may be cost-intensive and time-consuming, leading to a more localized and discipline-specific implementation and hindering a widespread use. The emergence of LMFMs could potentially revolutionize this field with their ability to comprehend and generate human-like text with a broad range of contexts and across multiple modalities, and LMFMs could significantly augment the capabilities of ITS, including cognitive tutors. For instance, their advanced contextual awareness could make cognitive tutors more effective in assisting with ill-defined problems.

Moreover, the possibility to easily generate pre-prompted chats and applications with LMFMs and to share these simple programs has opened up remarkable opportunities for both teachers and students, turning them into developers of specialized, user-friendly software applications. For example, recently OpenAI introduced a feature for their ChatGPT Plus users, termed GPTs^59^. These custom GPTs enable educators to rapidly create tools tailored for specific lessons, while students can develop applications to assist in their studies, for instance, for exam preparation. The ability to upload files with exercises and solutions ensures the precision of the GPT’s responses, a critical feature in educational environments^15^. The utilization of LMFMs in software development can potentially boost students’ programming self-efficacy and motivation, especially in specific scenarios. An example of this enhancement was observed during the incorporation of LMFMs into weekly programming exercises within programming education, as highlighted in a study by Yilmaz (2023)^62^. As a result, some scholars have recognized ChatGPT as an effective tool for imparting computational thinking skills^62^. Prior to the advent of LLMs, the development of software through low-code or no-code platforms was primarily embraced by individuals with some interest in computer science because of the significant time required to learn such a tool^63^. However, tools like LMFMs have a significantly reduced learning curve^64^. Therefore, this approach has the potential to expand its reach to individuals with less inclination towards programming.

#### Intelligent human-centered design process

Furthermore, LLM-powered platforms enable the creation of functional software without the necessity of explicitly specifying every intricate detail or meticulously planning the execution process. In this way, uncertain details and features of software that are often specified in a human-centered design process can be initially defined and later adapted by the LMFM. This reduction in cognitive effort paves the way for a widespread utilization across various domains, including recreational applications. Despite the reduced cognitive load compared to traditional programming, it is likely that the use of LMFMs to create software applications could enhance computational thinking skills. For instance, a user might initiate the creation of an application based on an LMFM without specifying every detail initially. During the initial use of the tool, they may encounter unexpected behaviors and subsequently refine and specify the previously overlooked details. It is possible that this iterative process of use could facilitate the development of a more precise ability to articulate intentions.

#### Collaborative development of complex software

Another significant opportunity lies in the use of advanced LLMs for complex software development. With the expanded context length of models like GPT-4 Turbo, LLMs can now consider and modify extensive amounts of source code, potentially encompassing 128k tokens which are more than 300 pages of text^65^. By sustaining a more extended context, users can develop intricate software, thereby augmenting the functionality and depth of the content they generate^64^. This feature may also be a boon for educational researchers who sometimes require sophisticated software platforms for their studies. Even with minimal programming knowledge, they can collaborate with LMFMs to express their needs (e.g., through drawings or speech) and develop complex platforms and user interfaces, a task that typically would require extensive funding and expertise. A notable example of collaborative programming involves the concept of Human-Bot Collaborative Architecting, with a specific emphasis on the integration of ChatGPT into human decision-making processes to streamline aspects of Architecture-Centric Software Engineering (ACSE). An initial case study within this domain explored the potential synergies between ChatGPT and architects’ decision-making, aiming to automate various facets of ACSE, as documented by Ahmad^66^. The authors also uncovered ChatGPT’s capacity to identify and address ethical, governance, and socio-technical considerations within this context^66^.

#### Code optimization

Furthermore, there are solutions that try to leverage the potential of simpler coding through LLMs. One example is CoPrompt, a system that assists programmers with prompt engineering in a collaborative context^67^. CoPrompt aids in prompt comprehension through detailed explanations, helping users understand and track the development of their programming tasks over time^67^.

Thus, *the integration of LMFMs in educational settings has huge potential to support teaching by offering personalized, up-to-date, and multimodal educational tools, enhancing lesson planning, enabling innovative teaching strategies, streamlining both extracurricular and administrative tasks, supporting professional development, and facilitating more effective assessments and feedback*.

## Challenges

### Challenges for learners

Students working with LMFMs face several challenges, each presenting unique complexities and considerations:

- **Output verification**: A critical challenge is verifying the accuracy of information generated by LLMs^68^. These models respond based on data patterns rather than genuine understanding, leading to potential inaccuracies or misleading information. LMFMs extend the capabilities of traditional LLMs by generating speech, images, such as diagrams, and videos, which introduces additional complexity in the output verification. For example, learning with LMFMs may require a learner to critically reflect on a visual representation that they may have difficulty understanding and extracting information from^33^. In this way, there is a risk that students might overly rely on the outcomes provided by LMFMs.
- **Verification of sources**: Another challenge arises from the difficulty of verifying the sources from which information is retrieved^69^. LLMs amalgamate vast amounts of data, making it hard to trace back the origin of specific pieces of information, which is crucial for reliability. For LMFMs, this issue is even more severe as the connected AI Models typically lack information of the training data set and how the output is generated by each model.
- **Maintaining an overview of AI models**: The EU’s General Data Protection Regulation gives users the right to know the basis of an algorithmic decision that significantly affects them^70^. The regulation thus enables the possibility of increased transparency in algorithmic decisions, among other things to avoid a “black-box society”^71^ and discrimination by algorithms^72^. However, this transparency is hindered by a lack of technical understanding of AI models^73^ and the challenge to maintain an overview of which AI models are involved in creating outputs of LMFMs. This awareness is key to understanding the limitations and capabilities of the responses they receive.
- **Potential segregation in society due to access costs**: Access to LMFMs can be expensive, potentially leading to segregation in society. This financial barrier might create exclusive learning groups that have access to LMFM tools, which may influence educational outcomes and further widening the learning gap between different socioeconomic groups.
- **Ethical issues and inherent biases**: LLMs and other AI tools may have a number of biases that could arise from various factors, such as unbalanced training datasets^74–77^. LMFMs raise significant ethical concerns, particularly regarding fair use. The intricacy and interconnectedness of these AI models can make it challenging to identify and rectify inherent biases. These models have the potential to mirror and amplify biases present in their training data, leading to the propagation of stereotypes or prejudiced viewpoints. This is a matter of considerable concern, particularly in educational settings.
- **Dependency on LMFMs**: Anderson and colleagues argue that artificial intelligence may erode human abilities, thus creating a dependence^78^. The ease of using LMFMs might lead to underdevelopment of certain skills. Students may become dependent on these tools for tasks they should be able to accomplish independently, thus reducing their effort and engagement in the learning process. Thus, there is a need for teachers and educational leaders to decide which human skills should remain part of the curriculum, and which can be dropped or reduced. It is necessary to identify which new skills are required and should be incorporated into the curriculum.
- **Overuse in learning situations**: There is a risk of LMFMs being overused in scenarios where students should be developing their own learning skills. This overuse may impede the development of critical thinking, research skills, and independent problem-solving abilities.
- **Severity of personal data leaks**: Concerns about personal data leaks are paramount^79^. Students may not be fully aware of which services process and store their data, what personal information is retained, who has access to it, and how it is protected.
- **Unwilling and unknowing involvement in plagiarism**: Although using an LMFM does not constitute plagiarism in itself, there is a risk that the content produced may inadvertently include plagiarized material. This raises concerns about academic integrity and the need for students to be vigilant about the originality of the content they submit.

Each of these challenges necessitates a careful and informed approach to using LMFMs in educational settings. Students and educators alike must be aware of these issues to effectively integrate these powerful tools into the learning process while mitigating potential risks. If learners are aware of the challenges and risks, the overarching distribution of applications based on LMFMs in the social environment of learners may lead to hesitation and fear of using them, as it may be difficult to discriminate between tools that are trustworthy and incorporate ethical standards and those that do not.

### Challenges for teachers and educators

Several key challenges for the educational community arise with the introduction of LMFMs in education:

- **Digital divide**: The implementation of LMFMs into teaching curricula carries the risk of widening the knowledge gap among learners due to an accessibility gap^80–82^. Not all educational institutions possess the financial and intellectual resources necessary to adopt and integrate such technologies. This could result in disparities in educational quality and opportunities, perpetuating existing inequalities among learners.
- **Perpetuating knowledge base**: The proliferation of use of LMFMs poses a risk of perpetuating existing knowledge bases and social biases^83,84^. LMFMs now draw on web- and training data released past the introduction of ChatGPT, which may also contain GPT-generated content, thus limiting exposure to genuinely new and diverse information. This potentially hinders the evolution of educational content and curricular materials.
- **Erosion of human relations**: The emphasis on AI-driven verbal communication tools may inadvertently contribute to the erosion of nonverbal communication competencies and social skills among learners and educators^2,85,86^. Overreliance on technology for communication could detract from the human interactions that foster social understanding and emotional skills. These skills include the ability to recognize emotions in others within the learning environment and to assist others in managing their emotions.
- **Reliability of information**: The integration of LMFMs makes it more challenging to assess the reliability of information, particularly for non-specialists and educators^87,88^. The ease of accessibility may obscure the need for critical evaluation, potentially leading to the dissemination of inaccurate or biased information in educational settings^83,84,89^. For instance, a teacher working outside their specialization may struggle to evaluate the credibility of the information provided by the sources they use to prepare for teaching. This could affect their ability to teach the subject effectively and confidently. Likewise, it may become more difficult for teachers to fairly assess the quality of student assignments.
- **Need for AI literacy**: The ease of access to LMFMs may create a distraction from the essential requirement to not only learn with but also about AI. There is a risk that the convenience of AI usage might overshadow the importance of developing a comprehensive understanding of its capabilities, limitations, and ethical considerations^90,91^.
- **Curriculum design and educational policies:** Educational institutions face the challenge of keeping pace with the rapid changes in LLM capabilities, necessitating constant adaptation of curricula and educational policies to ensure relevance and effectiveness^92,93^.

### Challenges for (educational) researchers

As AI tools such as LMFMs become more effective, accurate and multimodal, offering exciting opportunities for educational research and research in general, their widespread adoption must be approached with caution. It is essential to balance the use of AI with the preservation of rigorous, ethical, and insightful academic practices.

There is a risk of developing an overreliance on LMFMs, creating a dependency that could be problematic, such as:

- **Vulnerability in research methodologies:** Researchers could find themselves ill-equipped to adapt or continue their work without the help of AI, leading to vulnerability in their research methodologies.
- **Originality and authorship:** If researchers begin to rely predominantly on AI to write papers or proposals, the line between the researcher’s original ideas and AI-generated content may blur, raising questions about originality and authorship.
- **Transparency:** Even with the conversational nature of the data analysis tool, understanding how LMFMs arrive at certain conclusions or suggestions may be challenging. This lack of transparency can be a significant hurdle in research settings. Researchers may even be unable to explain the exact method of data analysis being used.
- **Over-trust:** It is likely that there is a risk of over-trusting the results and deductions drawn from LMFMs data analysis tools, which could lead to incorrect conclusions.
- **Bias in AI:** Despite advanced methods and benchmarks to mitigate bias in LMFMs and improve their alignment to social norms and goals^94–98^, bias in AI continues to be an issue^99,100^. AI algorithms could inadvertently perpetuate biases present in their training data, leading to biased research outcomes that further reinforce existing prejudices. A better understanding of the needs of LMFMs users in educational contexts, as well as human-centered evaluations of the underlying models, are both a necessity and a challenge, ensuring that such tools are both ethically aligned and transparent in their operations^101,102^. This is particularly concerning if researchers heavily rely on and trust AI, such as LMFMs.
- **Adaptability and limitations:** While LMFMs may offer improved adaptability due to, for example, their ability to handle extended context lengths^103,104^ or their improved vision capabilities^30^, there might still be limitations in fully meeting the diverse and specific needs of different research tasks and contexts. For example, during a systematic review, it is necessary to derive a balanced perspective on topics that may have been diversely displayed and discussed in multiple sources. Comparing and drawing conclusions from data presented in different documents, modalities and contexts may still be too complex for state-of-the-art LMFMs.
- **Ethical considerations:** Ensuring the ethical use of LMFM-driven data analysis tools, especially when dealing with sensitive educational data, is crucial. Researchers must navigate data privacy laws and ethical considerations. For example, under the EU AI Act, the use of generative AI in education would be subject to rigorous ethical and regulatory standards, focusing on risk assessment, transparency, data protection, and fairness^105^.
- **Resource requirements:** Utilizing advanced LMFMs often requires substantial computational and economical resources, which might not be readily available to all educational researchers.

Overall, the art of academic writing, critical thinking, and synthesis of information are at the core of research work. AI should be viewed by researchers as a supportive tool that enhances their work and empowers researchers to leverage its strengths, rather than a substitute for the indispensable skills they contribute.

### Challenges for developers in education

Despite the evident opportunities, the use of LMFMs in educational software development also presents several challenges, especially for novices, who may need more guidance and support to use them effectively. Some of the challenges include:

- **Complexity and accuracy**: ChatGPT-4.0, which can be used for Android app development, can handle simple tasks well, but may struggle with longer descriptions or more complicated functions. Developers often need to ask additional follow-up questions^106^. When given long descriptions, ChatGPT-4.0 sometimes generates incorrect or incomplete code. Although step-by-step guidance is beneficial for simpler tasks, it is not always sufficient for complete app development^106^. Novice developers frequently require extra guidance to resolve programming errors and learn additional steps that LMFMs may not provide, such as code placement and package imports^106^. An effective strategy for application development with ChatGPT involves breaking down the app into smaller functions and handling them separately, which necessitates more follow-up questions as complexity rises^106^. For novices, it is recommended to first learn basic coding through tutorials before using ChatGPT and to utilize specific commands for more accurate responses^106^. This approach underscores the potential for further research into ChatGPT’s application in more complex areas of app development^106^.
- **Adaptations for educational tool developers**: The rapid pace of development in LMFMs may present challenges for developers of educational tools. Keeping up with advancements, integrating them into tools, and understanding their potential roles in education are key issues. Technical challenges and resource-intensity add to the complexity.
- **Fine-Tuning in education**: A significant challenge in leveraging MLLMs for educational purposes lies in the process of fine-tuning. Fine-tuning these models to suit specific educational needs and contexts requires a substantial amount of relevant and high-quality data. However, obtaining such data in the educational field is often a hurdle, primarily due to privacy concerns, limited availability of diverse educational content, and the proprietary nature of many educational resources. Hence, scarcity of data can hinder the ability to tailor these AI models effectively for educational applications. It also raises questions about the generalizability and adaptability of the models to diverse educational settings and learner needs. Overcoming these challenges requires not only innovative approaches to data collection and sharing in the educational domain, but also the employment of methods for data augmentation and generation^107–109^.
- **Consistency and ethics**: Studies on Architecture-Centric Software Engineering (ACSE) using ChatGPT highlight several concerns^66^. The varied responses from ChatGPT can affect the consistency in architecting, and the content generated may raise legal issues regarding intellectual property as well as ethical concerns^66^. Biases in ChatGPT’s training data might also influence the quality of outputs^66^. To address these challenges, further empirical studies are necessary to validate the productivity and efficacy of ChatGPT in various contexts and teams^66^.
- **Access**: An additional challenge is the potential restriction of access to LMFMs, which may be limited to those who can afford them, especially if no viable alternatives from large companies exist. This can be seen in the restricted access to GPTs generated by OpenAI’s users. The reliance on proprietary platforms raises concerns about inclusivity and equitable access to advanced educational technology, potentially creating a divide between those who can and cannot afford these tools^91^. Ensuring broad and equitable access to these transformative technologies is crucial to avoid exacerbating existing inequalities in education.

## Mitigation strategies

To effectively integrate LMFMs in education, it is critical for the educational community to adopt multifaceted risk mitigation strategies. Emphasizing AI as a supplementary tool in teaching is paramount, ensuring it complements rather than replaces human teaching.

Educators and researchers alike must navigate these advancements responsibly, upholding ethical standards and maintaining rigorous scholarly practices. Researchers need to engage in ongoing education regarding LMFM advancements, rigorously uphold ethical standards, and transparently document AI utilization, fostering responsible integration within scholarly pursuits. Educators need comprehensive training in AI literacy, ethical usage, data privacy, and fact-checking to critically assess AI-generated content. Likewise, engaging parents as part of the educational community in understanding LMFMs’ educational impact is essential to create responsible LMFM-integrated learning environments and to foster their long-term acceptance.

For researchers delving into the integration of LMFMs into academic inquiry, several proactive strategies emerge as pivotal:

- **Systematic evaluation of AI limitations:** Instituting a regime of systematic and field-specific assessments to ascertain the bounds and constraints of the knowledge of LMFMs, which can enable researchers to discern where human expertise is indispensable and where AI augmentation is beneficial.
- **Continuous engagement with LMFM advancements:** Maintaining an ongoing commitment to understanding LMFM developments ensures researchers remain well-versed in the evolving landscape of these technologies.
- **Transparent documentation of LMFM utilization:** Transparently documenting the use of LMFM tools throughout the research process fosters transparency and integrity in scholarly pursuits.
- **Critical assessment and cross-referencing:** Embracing a critical mindset entails consistently comparing LMFM-generated outputs with established resources, upholding the integrity of scholarly claims.
- **Diversified LMFM training data and bias mitigation:** Advocating for diversified training data and employing bias detection and mitigation techniques helps in fostering fairer and more inclusive research outcomes.

Simultaneously, within educational frameworks, strategies to integrate AI tools responsibly among teachers and students are fundamental. In the design of curricula incorporating AI in general, and LMFM in particular, a focus on AI literacy is essential. Making it a core part of the curriculum is necessary for both teachers and students, providing a clear understanding of AI’s and LMFM’s capabilities and limitations. Additionally, curricula should include modules on fact checking and ethical considerations of AI, aiming to develop students’ critical evaluation skills of AI-generated content. Finally, creating assessment strategies that align with learning objectives in LMFM-enhanced environments is crucial, ensuring that these strategies effectively gauge student understanding and the application of critical thinking in the context of LMFMs.

In educational policies and structures, mitigating the challenges posed by LMFMs involves several key strategies. Designing teacher training systems that adapt to rapid changes in educational technology is crucial. This includes prolonged phases of continual further qualification, enabling educators to stay informed about the evolving AI landscape. Addressing the digital divide is also essential; formulating policies and prioritizing resource allocation are necessary steps to ensure equitable access to AI technologies for all students, thereby creating a level playing field. Monitoring AI developments for biases is important to maintain fairness in educational applications, which requires regular policy reviews and updates. Additionally, promoting research into the pedagogical implementation of LMFMs in educational settings is vital. This helps in developing a knowledge base that informs effective teaching practices with LMFMs, contributing to the creation of evidence-based educational strategies.

### Open-source vs. proprietary LMFMs

Open-source vs. proprietary LMFMs: In the long term, the choice between open-source and proprietary LMFMs will be critical, as open-source LMFMs are more accessible and cost-effective, offering customization, transparency, and community support, which are beneficial for collaborative learning and innovation. Open-source models will allow educators and researchers to tailor models to specific needs and ensure ethical alignment, but can pose challenges in data security. However, this will also require more advanced technical skills around AI literacy, model fine-tuning, and quality assurance. With further technological advancements, the choice between open-source and proprietary LMFMs may not only impact the future of learning but also pave the way for a new era of digitally empowered, inclusive education.

## Conclusion

The emergence of LMFMs like ChatGPT-4-Turbo and Gemini represents a significant shift in the landscape of education, offering unparalleled opportunities for personalized learning, enhanced engagement, and innovative educational methodologies. In our perspective, it is important to understand LMFMs as tools with the potential to transform how we learn, teach, and conduct educational research by enabling more interactive, tailored, and efficient educational environments that teachers and students can personalize without the need for programming skills. However, it is important to understand the new challenges that arise with this technological revolution and not to replace human teachers, developers, and researchers as they are central to understanding, communicating, and mitigating the risks from LMFMs. Therefore, it is also relevant to keep the human in the loop, for instance, as a responsible teacher and/or an increasingly self-directed learner.

For learners, LMFMs present opportunities for enhanced learning experiences through personalization, barrier-free interaction, and support in various learning activities. Yet, there are challenges such as verifying the accuracy of LLM outputs, ethical issues, potential biases in AI models, and the risk of overreliance, which could hinder critical thinking and independent learning.

Educators stand to benefit from LMFMs through innovative teaching strategies, lesson planning, and professional development. However, they also face challenges like the digital divide, erosion of human relations, reliability of information, and the need for AI literacy. These challenges necessitate a reevaluation of teaching methodologies and the development of new competencies.

Educational researchers are presented with advanced data analysis capabilities and tools for creating multimodal assessment and learning material. However, they must exercise caution regarding overreliance on LMFMs, maintain transparency, and address ethical considerations.

Developers in education can now more easily create intelligent learning applications and engage in human-centered design processes. Nevertheless, they face issues related to the complexity and accuracy of LMFMs, consistency in outputs, ethical concerns, and access limitations.

To mitigate these challenges, a multifaceted approach is needed. For the educational community, emphasizing AI as a supplementary tool, providing comprehensive AI literacy training for educators and learners, and engaging parents in understanding the impact of LMFMs are essential. Researchers should systematically evaluate AI limitations, engage continuously with advancements, document LMFM utilization transparently, and employ diversified training data for bias mitigation. In educational policy and curriculum design, focusing on AI literacy, developing critical evaluation skills, and formulating equitable access policies are crucial. Moreover, choosing open-source over proprietary LMFMs can be crucial for a more flexible and community-centered future of education; however, the advantages related to accessibility, independent customization, and ethics require higher levels of technical skills and regulatory expertise.

Ultimately, while LMFMs offer a revolutionary path forward in education, balancing their benefits with the inherent challenges requires careful consideration, ethical awareness, and a commitment to ongoing adaptation and learning from all involved groups, i.e., teachers, students, educational researchers, and developers. The joint efforts of all involved groups will ensure that the integration of LMFMs in educational settings is done responsibly, ethically, and effectively, contributing to a future where technology enhances the educational experience.
